# Ketones prevent synaptic dysfunction induced by mitochondrial respiratory complex inhibitors

**DOI:** 10.1111/j.1471-4159.2010.06728.x

**Published:** 2010-04-02

**Authors:** Do Young Kim, Johana Vallejo, Jong M Rho

**Affiliations:** * Barrow Neurological Institute and St. Joseph’s Hospital & Medical Center, Phoenix, Arizona, USA; † Department of Physiology, Midwestern University, Glendale, Arizona, USA

**Keywords:** ATP, ketones, mitochondrial respiratory complex, neurodegenerative disease, oxidative stress, synaptic transmission

## Abstract

Ketones have previously shown beneficial effects in models of neurodegenerative disorders, particularly against associated mitochondrial dysfunction and cognitive impairment. However, evidence of a synaptic protective effect of ketones remains lacking. We tested the effects of ketones on synaptic impairment induced by mitochondrial respiratory complex (MRC) inhibitors using electrophysiological, reactive oxygen species (ROS) imaging and biochemical techniques. MRC inhibitors dose-dependently suppressed both population spike (PS) and field potential amplitudes in the CA1 hippocampus. Pre-treatment with ketones strongly prevented changes in the PS, whereas partial protection was seen in the field potential. Rotenone (Rot; 100 nmol/L), a MRC I inhibitor, suppressed synaptic function without altering ROS levels and PS depression by Rot was unaffected by antioxidants. In contrast, antioxidant-induced PS recovery against the MRC II inhibitor 3-nitropropionic acid (3-NP; 1 mmol/L) was similar to the synaptic protective effects of ketones. Ketones also suppressed ROS generation induced by 3-NP. Finally, ketones reversed the decreases in ATP levels caused by Rot and 3-NP. In summary, our data demonstrate that ketones can preserve synaptic function in CA1 hippocampus induced by MRC dysfunction, likely through an antioxidant action and enhanced ATP generation.

Mitochondria play pivotal roles in cellular homeostasis through a multiplicity of actions, including – but not limited to – ATP generation, Ca^2+^ buffering, and regulation of reactive oxygen species (ROS). Importantly, the mitochondrial respiratory complex (MRC), which comprises five distinct multimeric units lining the mitochondrial inner membrane, is critical in the formation of ATP (Foster *et al.* 2006; Keating 2008). However, when there is excessive electron flux or shunting through the MRC (i.e., through disruption of redox reactions or increases in the mitochondrial membrane potential, ΔΨ), there is increased ROS production and inhibition of ATP synthesis. Such an imbalance in ATP and ROS generation by the MRC is believed to contribute to the pathogenesis of neurodegenerative diseases (ND) (Cassarino and Bennett 1999; Mancuso *et al.* 2006; Kang *et al.* 2007; Petrozzi *et al.* 2007).

Mitochondrial respiratory complex inhibitors such as rotenone (Rot) and 3-nitropropionic acid (3-NP) can induce neuropathological changes in both *in vivo* and *in vitro* models of Alzheimer’s disease (AD), Parkinson’s disease (PD), Huntington’s disease and amyotrophic lateral sclerosis, comparable to that seen in humans (Alexi *et al.* 1998; Saybasili *et al.* 2001; Kweon *et al.* 2004; Zhao *et al.* 2006). As synaptic transmission is highly dependent on mitochondrial products such as ATP and ROS (Keating 2008), it was reasoned that synaptic integrity under such pathological conditions would be compromised. In support of this, MRC dysfunction has been reported in patients with AD and Huntington’s disease who exhibit cognitive/memory impairment and failure of energy metabolism (Huber *et al.* 1986; Davies and Ramsden 2001; Panov *et al.* 2005). Further, it has been shown that MRC inhibitors impair field potential activity in the rat hippocampus, the primary locus of memory and cognitive consolidation (Costa *et al.* 2008). Collectively, these observations suggest that maintenance of mitochondrial respiration may be essential for the preservation of synaptic integrity. However, direct evidence linking hippocampal synaptic suppression arising from MRC dysfunction and an imbalance in ATP/ROS production has not been forthcoming.

The ketogenic diet (KD) is a remarkably effective non-pharmacological treatment for patients with intractable epilepsy, and is designed to reproduce the biochemical changes seen upon fasting, as well as during suckling periods in immature animals (Kim do and Rho 2008; Prins 2008). Further, there is mounting evidence that the KD and some of the metabolic substrates elaborated by the KD exert neuroprotective activity. Specifically, the two major ketones, (R)-(−)-3-hydroxybutyric acid (BHB) and acetoacetate (ACA), have previously been shown to enhance neuronal viability in models of hypoxic-ischemic brain injury, and other experimental models of ND (Dardzinski *et al.* 2000; Kashiwaya *et al.* 2000; Suzuki *et al.* 2001; Veech 2004; Masuda *et al.* 2005). Furthermore, KTX-0101, a synthetic BHB ester-linked polymer has shown promise in early clinical trials as a potential treatment for ameliorating cognitive impairment in Alzheimer’s disease (Smith *et al.* 2005). And more recently, Axona (ACC-1202) – a medical food that is metabolized into ketone bodies–was approved by the US FDA for the treatment of moderate Alzheimer’s disease.

However, despite mounting evidence for the potential beneficial effects of ketones in various ND, whether these substrates can preserve synaptic function under conditions of MRC dysfunction remains unclear. To address this issue, we examined whether MRC dysfunction induced by either Rot or 3-NP resulted in hippocampal synaptic impairment using cellular electrophysiological methods, and then asked whether this was because of changes in ATP and/or ROS production using fluorescence imaging and biochemical assay techniques. We found that ketones exerted a protective effect in our model, likely through either antioxidant modulation and/or ATP generation.

## Materials and methods

### Preparation of brain slices

All animal handling protocols were approved by the Institutional Animal Care and Use Committee at the Barrow Neurological Institute and St. Joseph’s Hospital & Medical Center. Transverse hippocampal slices (400 μm) were prepared from brains of 4- to 7-week-old Sprague-Dawley rats. Following decapitation, the whole brain was rapidly isolated and submerged in ice-cold oxygenated physiological saline (composition in mmol/L: 124 NaCl, 1.8 MgSO_4_, 4 KCl, 1.25 NaH_2_PO_4_, 26 NaHCO_3_, 2.4 CaCl_2_, and 10 d-glucose; pH = 7.4). Slices were cut using a standard vibratome (The Vibratome Company, St. Louis, MO, USA), and then transferred to an incubation chamber containing physiological saline bubbled with 95% O_2_/5% CO_2_ at 35°C for 1 h.

### Electrophysiology

Each slice was transferred into a submersion-type recording chamber attached to an Axioskop FS2 microscope (Carl Zeiss Microimaging, Inc., Thornwood, NY, USA) and superfused with warm (35 ± 1°C) physiological saline at a rate of 3–4 mL/min before the start of each experiment. Population spikes (PS) were evoked by stimulation of Schaffer collaterals using a bipolar concentric electrode and recorded in the stratum pyramidale of cornu Ammonis (CA) 1 with a recording electrode (2–6 MΩ tip resistance, backfilled with 2 mmol/L NaCl) connected to a Multiclamp 700A amplifier and digitized with a Digidata 1322A interface (Axon Instruments, Union City, CA, USA). Stimulation parameters were as follows: pulse duration (100 μs), stimulus intensity (20–100 μA) set to 50% of the maximal PS amplitude. After obtaining a stable evoked PS, changes in PS amplitude during test compound application were digitally stored for later off-line analysis. Upon Schaffer collaterals stimulation, excitatory post-synaptic potentials (field potential) were induced at a control test frequency of 0.05 Hz (0.1 ms, 20–100 μA) from the stratum radiatum of CA1. From a standard input-output curve (i.e., stimulus intensity vs*.* field potential amplitude), the baseline field potential amplitude (over 1 mV) was set to 50% of the maximum responses. Recorded field potential data were filtered at 3 Hz, sampled at 10 kHz using pClamp, and analyzed using Clampfit (Axon Instruments). All electrophysiological experiments were conducted following stabilization of the baseline amplitude for both PS and field potentials during a 30 min physiological saline infusion.

### Reactive oxygen species imaging

2′,7′-Dichlorofluorescein diacetate (H_2_-DCFDA), which oxidizes to the fluorescent 2′,7′-dichlorofluorescein (DCF), was used to measure intracellular H_2_O_2_ levels. H_2_-DCFDA was directly injected into CA1 pyramidal neurons via the patch pipette under whole-cell recording conditions using a protocol modified from Bao *et al.* (2005). Briefly, individual CA1 pyramidal neurons were visualized with differential interference contrast optics under infrared illumination. Recording electrodes (4–7 MΩ) were pulled from borosilicate glass and backfilled with a mixture of patch pipette solution (composition in mmol/L: 140 K^+^-gluconate, 10 HEPES, 2 MgCl_2_, 1 CaCl_2_, 1 EGTA, and 2 K_2_ATP, pH = 7.25) and H_2_-DCFDA (7 μmol/L). All electrophysiological experiments were performed using the tight-seal, whole-cell configuration. Resting membrane potential and membrane conductance of CA1 pyramidal neurons were determined in current-clamp mode after a minimum 20 min incubation period with physiological saline. Fluorescence was measured with a Stallion 2 imaging system (Carl Zeiss Microimaging, Inc.) using an Axiocam camera mounted to an Axioskop FS2 microscope. After a 20-min period of equilibration, images were recorded every 20 s under fluorescence (exposure time, < 400 ms). Fluorescence intensity after drug application was analyzed using a slide book (Intelligent Imaging Innovation, Santa Monica, CA, USA). Neuronal fluorescence was normalized to background (i.e., cell-free area) fluorescence. In separate ROS imaging experiments, brain slices were incubated in physiological saline mixed with 50 μmol/L dihydroethidium (DHE) for 30 min and then imaged to detect changes in fluorescence intensity of CA1 pyramidal neurons after either Rot or 3-NP application. Captured images were acquired under fluorescence (exposure time, 50–100 ms) every 30 s.

### Measurement of catalase activity

Brain slices were prepared as described above and treated with either H_2_O_2_ (2 mmol/L) or a ketone cocktail (BHB plus ACA, 1 mmol/L each), or simultaneously exposed to both ketones and H_2_O_2_. After treatment, tissue samples (400 μm thickness) were micro-dissected from the CA1 region using 18-gauge needles to ensure uniformity of size. Each assay sample (1 mg total tissue) consisted of three individual micro-dissected CA1 sections, combined and weighed in a microcentrifuge tube. Samples were homogenized in 25 μL of 0.1 mol Tris–HCl and then sonicated for 10 min. After sonication, each sample was subjected to centrifugation (5 min at 14 000 *g*, 4°C) and the supernatant was collected for analysis. All samples were mixed with reagents from a commercial catalase assay kit (Amplex Red Catalase Assay Kit; A22180; Molecular Probes, Eugene, OR, USA) and subjected to incubations according to the manufacturer’s instructions. Light absorbance from the oxidation product, resofurin, was detected at 560 nm using a microplate reader (Bio-Rad Model 680, Hercules, CA, USA). Each sample was normalized to a control (i.e., no catalase) and then compared to a catalase standard curve obtained from serial dilutions of a catalase stock solution provided with the kit.

### ATP assay

Tissue samples were micro-dissected from the CA1 region of rat brain slices (*n*= 8–10 in each experimental group from three to four rats) using 18-gauge needles under a microscope. Each sample was weighed in a microcentrifuge tube and then sonicated in 5% trichloroacetic acid (100-fold dilution; volume/weight) to block ATP degradation. After 10 min of sonication, samples were subjected to centrifugation (2 min at 14 000 *g*, 4°C). For each sample, 10 μL of supernatant was diluted 1000-fold in phosphate-buffered saline and then mixed with the Enlighten ATP assay kit reagents (Promega, Medison, WI, USA) according to the manufacturer’s instructions. Light output from each reaction was captured in a TD-20/20 Luminometer (Turner Biosystems, Sunnyvale, CA, USA) and then compared to an ATP standard curve obtained from serial dilutions of an ATP stock solution provided with the kit.

### Drugs

All chemicals used in this study were purchased from Sigma-Aldrich (St. Louis, MO, USA), unless otherwise stated. The following agents were used: BHB, sodium salt; ACA, lithium salt; catalase (from bovine liver); 3-nitropropionic acid (3-NP); Mn(III) tetrakis (4-benzoic acid) porphyrin (MnTBAP; Calbiochem, La Jolla, CA, USA) and glutathione monoester (GSHMEE). These chemicals were directly dissolved in physiological saline. Rot was added to physiological saline as a 10 mmol/L stock solution dissolved in dimethylsulfoxide (DMSO). H_2_-DCFDA or dihydroethidium (Molecular Probes) was dissolved in DMSO and then mixed with the applied solution to reach a final DMSO concentration of below 0.01%.

### Statistics

Numerical data are expressed as the mean ± SEM for *n* slices. Either a Student’s *t*-test or anova was performed to assess significant differences and variance among different experimental groups. Significance was set at *p*< 0.05.

## Results

### Ketones protect synaptic transmission against rotenone in a dose-dependent manner

**Figure 1:**
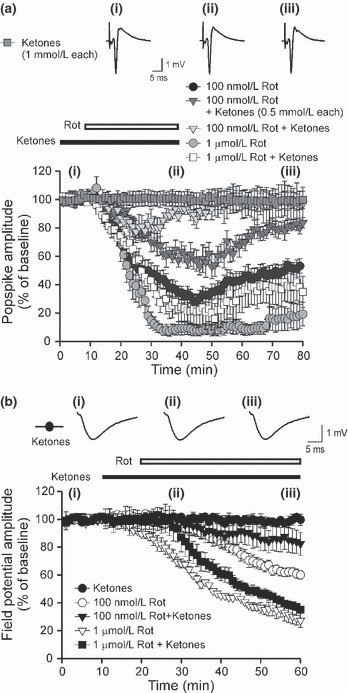
Ketones suppress rotenone (Rot)-induced hippocampal synaptic suppression in a dose-dependent manner. (a) Time-course of the mean population spike (PS) amplitude after application of either 100 nM or 1 μM rotenone (Rot) with or without ketones. Either 100 nmol/L (•) or 1 μmol/L (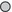) Rot strongly depressed the PS amplitude during and after Rot application. Ketones alone 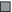, Each 1 mmol/L) did not alter the PS amplitude. Pre-treatment with ketones fully reversed the synaptic suppression seen with 100 nmol/L Rot (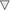), but this was partially restored with 1 μmol/L Rot (□). In each condition where 0.5 mmol/L ketones were tested against 100 nmol/L Rot (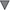), ketones induced partial protective effects compared to 1 mmol/L ketones. (b) Changes in the mean field potential amplitude before, during, and after either 100 nmol/L or 1 μmol/L Rot with or without ketones. Rot slightly suppressed the field potential amplitude compared to the change in PS amplitude after Rot treatment. Both 100 nmol/L and 1 μmol/L Rot-induced field potentials were partially restored by ketone application. Representative traces of PS or field potentials at respective time-points (i, ii, iii) are depicted in the traces above. The horizontal line in this and following figures indicates the drug infusion period. Data are expressed as mean ± SEM.

Previous studies have suggested that rotenone (Rot)-induced neuronal injury may be a consequence of mitochondrial ROS production and ATP depletion (Sherer *et al.* 2003; Bao *et al.* 2005; Zhao *et al.* 2006). Although Rot-induced pathological changes have been associated with suppression of synaptic transmission, a direct demonstration of this has been elusive. We first tested the effect of Rot, a MRC I inhibitor, on the evoked population spike (PS) in CA1 hippocampus. Upon exposure to 1 μmol/L Rot (10 slices from six rats), the PS amplitude was strongly suppressed to 9.2 ± 4.9% and 16.0 ± 8.3% of baseline within 30 min after Rot application and 40 min after washout, respectively (Fig. 1a). With 100 nmol/L Rot (16 slices from eight rats), the PS amplitude decreased to 34.2 ± 4.6% of baseline after 30-min incubation, and then increased to 53.1 ± 5.0% of baseline after 40 min of washout (Fig. 1a).

Next, to determine whether ketones alone affected the evoked PS, we tested the effects of a cocktail of BHB and ACA (each 1 mmol/L or 0.5 mmol/L). These concentrations were chosen because previous studies demonstrated that CSF concentrations of BHB and ACA were approximately 0.3–0.4 mmol/L after KD treatment in children, and because brain BHB levels had previously been detected in the range of 0.5–1.6 mmol/L (Nordli and De Vivo 1997; Thio *et al.* 2000). In addition, physiological blood concentrations of ketones were observed to be about l mmol/L during the suckling period in immature rodents or during treatment with the KD in humans (Nordli and De Vivo 1997; Izumi *et al.* 1998; Thio *et al.* 2000).

As expected, the PS amplitude did not change during ketone cocktail infusion and after washout (Fig. 1a). However, ketones (each 1 mmol/L) strongly rescued the PS depression induced by 100 nmol/L Rot; PS amplitudes measured 93.9 ± 3.8% and 97.0 ± 11.7% of baseline after 30 min of ketones plus Rot application and 40-min washout, respectively (nine slices from five rats; Fig. 1a). While the PS amplitude in response to 100 nmol/L Rot was partially restored by a cocktail consisting of BHB and ACA (each 0.5 mmol/L), this effect was less than that observed when each ketone applied independently at 1 mmol/L (eight slices from four rats; Fig. 1a). When slices were exposed to 1 μmol/L Rot, ketones exerted only modest effects, as compared to 1 μmol/L Rot alone (nine slices from four rats; Fig. 1a).

A recent study reported that either 30 or 100 μmol/L Rot strongly suppressed the corticostriatal field potential amplitude (Costa *et al.* 2008), but the amplitude was only slightly diminished by 1 μmol/L Rot. Interestingly, exposure of CA1 hippocampus to either 1 μmol/L or 100 nmol/L Rot in our hands elicited suppression of the field potential amplitude in a dose-dependent manner; the field potential amplitude decreased to 60.0 ± 3.0% and 27.2 ± 4.9% of baseline after 40 min of Rot application, respectively (14 slices from six rats per each study; Fig. 1b). Consistent with a lack of an effect of ketones on the PS, the field potential amplitude did not change significantly during a 50-min application of ketones alone. However, ketones applied together with either 1 μmol/L or 100 nmol/L Rot led to partial recovery of the suppressed field potential amplitude: 82.4 ± 8.2% and 35.1 ± 2.5% of baseline after 40 min of ketones plus Rot application (13 slices from five rats per each study; Fig. 1b).

### Ketones protect against 3-nitropropionic acid-induced synaptic suppression

**Figure 2:**
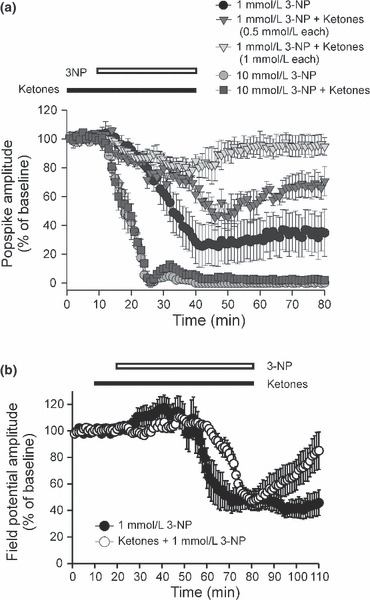
Ketones mitigate synaptic suppression induced by the mitochondrial complex II inhibitor [3-nitropropionic acid (3-NP)]. (a) Either 1 mmol/L (•) or 10 mmol/L (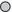) irreversibly suppressed the PS amplitude in CA1 hippocampus. Pre-treatment with ketones did not produce a synaptic protective effect against 10 mmol/L 3-NP (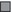), whereas ketones significantly reduced PS suppression induced by 1 mmol/L 3-NP (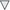), and then restored the PS amplitude to basal levels after a 20-min washout. Lower concentrations of ketones (0.5 mmol/L each) were partially protective (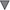). (b) During the initial period of 1 mmol/L 3-NP treatment (○), the mean field potential amplitude was slightly increased. After 30 min, the field potential showed dramatic decay and then did not change during wash-out. While pre-treatment with ketones slowly decreased the field potential amplitude compared to 1 mmol/L 3-NP alone, no beneficial effect occurred after 60 min of co-application. However, ketones significantly enhanced the recovery of the field potential amplitude after wash-out.

In subsequent experiments, we tested whether inhibition of MRC II activity would also result in impairment of hippocampal synaptic transmission. In slices exposed to 0.3 mmol/L 3-NP, the PS amplitude actually increased 110.2 ± 8.2% and 110 ± 7.1% of baseline within 30 min of 3-NP application and 40 min after washout, respectively (data not shown, eight slices from four rats). Conversely, either 1 mmol/L or 10 mmol/L 3-NP strongly decreased the PS amplitude to 28.4 ± 14.8% vs*.* 0.09 ± 0.1% and 34.8 ± 16.3% vs. 0.29 ± 0.12% of baseline at 30 min after the start of drug application and 40 min after washout, respectively (12 slices from five rats; Fig. 2a). When the two ketones were administered with 1 mmol/L 3-NP, the PS amplitude recovered to 83.5 ± 4.6% and 94.5 ± 6.0% of baseline after 30 min of ketones plus 1 mmol/L 3-NP application and 40 min post-washout, respectively (13 slices from six rats; Fig. 2a). Similar to our observations with Rot, either ketone alone (0.5 mmol/L) only partially protected the PS amplitude against 1 mmol/L 3-NP (eight slices from four rats; Fig. 2a). However, ketone-mediated synaptic protective effects were completely abolished by 10 mmol/L 3-NP (12 slices from five rats; Fig. 2a).

Extending these findings, we measured the changes in field potential amplitude caused by 1 mmol/L 3-NP with and without ketone application. The field potential amplitude initially increased, and then decreased after 60 min of 3-NP exposure; the amplitude changed to 46.5 ± 6.1% of baseline (11 slices from five rats; Fig. 2b). The smaller field potential induced by 3-NP changed irreversibly during the 30-min washout (45.8 ± 9.5% of baseline). While ketone application resulted in a slight delay of 3-NP-induced synaptic suppression, the field potential amplitude after 60 min of ketones plus 3-NP was not significantly different than with 3-NP alone (Fig. 2b). Surprisingly, the suppressed field potential after co-application of ketones and 3-NP recovered to 85.0 ± 14.0% of baseline after washout. Overall, these results indicated that ketones provide a functional protective effect against both MRC I and II inhibition in CA1 hippocampus.

### Ketones mimic antioxidant effects

**Figure 3:**
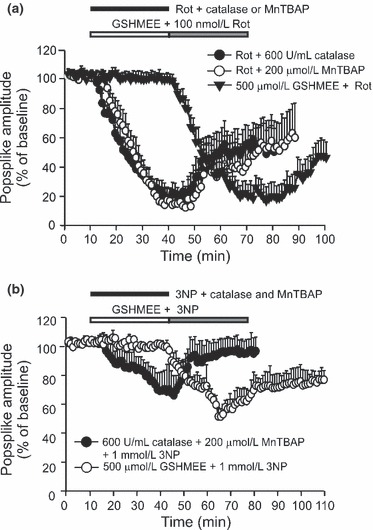
Effects of antioxidants on hippocampal synaptic suppression by mitochondrial respiratory complex (MRC) inhibitors. (a) Neither 600 U/mL catalase (•) nor 200 μmol/L MnTBAP (○) blocked the synaptic suppression caused by 100 nmol/L Rot. Pre-treatment with cell-permeable 500 μmol/L GSHMEE (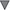) alone also had no effect on Rot-induced PS suppression. (b) Co-application of a cocktail of 600 U/mL catalase and 200 μmol/L MnTBAP with 1 mmol/L 3-NP resulted in recovery of the PS amplitude. Similarly, pre-treatment with GSHMEE also restored the PS amplitude against 3-NP application.

Earlier, we showed that ketones induce neuronal/synaptic protection against oxidative stress via suppression of ROS (Kim *et al.* 2007; Maalouf and Rho 2008). Thus, we reasoned that the synaptic protective effect of ketones against MRC dysfunction might involve an antioxidant mechanism. To address this, we first compared the effect of antioxidants – such as catalase (a direct scavenger of H_2_O_2_), MnTBAP (a superoxide scavenger), and GSHMEE, which are subsequently converted intracellularly to glutathione – under conditions of ketone-induced protection against MRC inhibitor impairment of the PS. Unexpectedly, neither catalase (600 U/mL; 10 slices from five rats) nor MnTBAP (200 μmol/L; 10 slices from five rats) affected the synaptic suppression induced by 100 nmol/L Rot (Fig. 3a). Next, to rule out the possibility that the negative effect of co-application of either catalase or MnTBAP with Rot was confounded by a delay in intracellular action, 500 μmol/L GSHMEE was pre-infused for 30 min, and then subsequently co-applied with Rot in hippocampus. Like catalase, GSHMEE failed to exert any beneficial effect against Rot-induced synaptic suppression (10 slices from five rats; Fig. 3a), supporting the notion that the synaptic protective effect of ketones against 100 nmol/L Rot are ROS-independent.

In contrast, co-application of catalase and MnTBAP restored the PS depression induced by 1 mmol/L 3-NP; the PS amplitude measured 79.9 ± 11.0% and 93.4 ± 8.7% of baseline at 30 min after the start of co-application and 40 min after washout, respectively (12 slices from five rats; Fig. 3b). Similar to the protective effects of catalase and MnTBAP, GSHMEE suppressed 3-NP-induced synaptic impairment; the PS amplitude measured 67.1 ± 8.7% and 78.0 ± 7.2% of baseline at 30 min after at 30 min after the start of co-application and 40 min after washout, respectively (12 slices from five rats; Fig. 3b). These findings indicated that 3-NP-induced synaptic suppression partially involves ROS generation, and indirectly suggest that ketones-mediated synaptic protection may involve enhanced antioxidant capacity. To explore this possibility, we examined the effects of ketones on standard measures of antioxidant activity.

### Ketones enhance catalase activity

**Figure 4:**
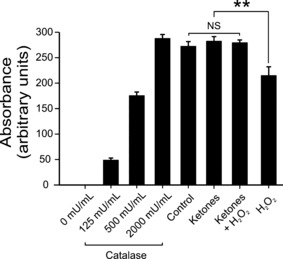
Ketones enhance catalase activity under conditions of oxidative stress. Calibration curve demonstrating an increase in arbitrary unit values as catalase dose increases. Similar to the effects seen with 2000 mU/mL of catalase alone, no differences were seen between physiological saline, ketones, and ketones plus 2 mmol/L H_2_O_2_ groups after 3 h of treatment. Conversely, H_2_O_2_ alone significantly decreased the level of catalase compared to the above three groups (**, *p* < 0.01). NS, not significant.

Previous studies have suggested that the therapeutic effects of either the KD or ketones may be because of enhanced glutathione peroxidase activity, and oxidation of Co-enzyme Q and NADH (Veech *et al.* 2001; Ziegler *et al.* 2003). Intriguingly, developmental increases in glutathione peroxidase activity facilitated resistance to H_2_O_2_ toxicity via activation of catalase in rat oligodendrocytes (Baud *et al.* 2004). To determine whether ketones affect antioxidant capacity, we directly measured changes in levels of this enzyme in CA1 hippocampus after exposure to H_2_O_2_ with or without ketones. A calibration curve relating enzyme activity to absorbance of catalase (on an arbitrary scale) was first established: 0, 125, 500, and 2000 mU/mL catalase corresponded to 0 ± 0, 49.3 ± 3.5, 176 ± 6.6 and 288 ± 7.2 absorbance units, respectively (Fig. 4). Catalase levels of physiological saline (control), ketones and ketones plus 2 mmol/L H_2_O_2_-treated CA1 hippocampus were 273 ± 8.8, 284 ± 8.5 and 281 ± 5.1, respectively (six samples each from four rats; Fig. 4); no significant differences among these groups were seen. In contrast, H_2_O_2_ application alone (220 ± 10; seven samples each from four rats; Fig. 4) significantly decreased catalase levels compared to these three groups (*p*< 0.01). These data indicated that the synaptic protective effect of ketones may partially involve enhanced antioxidant capacity against ROS-induced oxidative stress.

### Ketones suppress ROS generation induced by MRC inhibition

**Figure 5:**
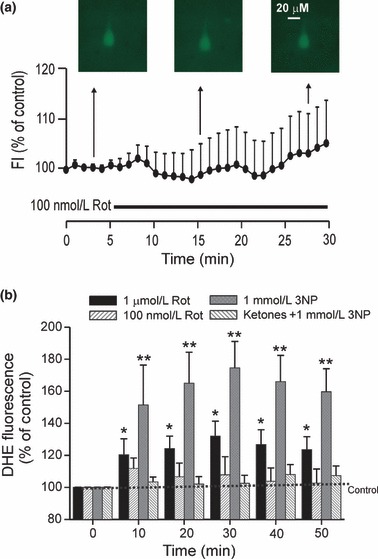
Changes in ROS levels after MRC inhibition. (a) No changes in DCF fluorescence intensity (FI) were seen in CA1 pyramidal neurons exposed to 100 nmol/L Rot over 30 min. (b) In the control group, pyramidal neurons were perfused with aCSF solution containing dithroethidium (DHE), and only a slight increase in DHE intensity was seen over time. Rot evoked a dose-dependent increase in DHE fluorescence intensity, but 100 nmol/L Rot did not evoke a significant change in DHE intensity compared to control. One mmol/L 3-NP prominently enhanced reactive oxygen species (ROS) among the three groups, whereas ketone application largely suppressed ROS generation induced by 3-NP. Values represent mean ± SEM. One way anova followed by Tukey test; *, *p* < 0.05; **, *p* < 0.01.

Although our initial electrophysiological data suggested that Rot-induced synaptic suppression is ROS-independent, it is well known that a common feature in the early stages of mitochondrial dysfunction is ROS generation (Saybasili *et al.* 2001; Kudin *et al.* 2004). To clarify our initial findings, we directly measured the change in ROS levels in CA1 pyramidal neurons exposed to 100 nmol/L Rot using DCFDA, a cell-permeant indicator which is oxidized to the fluorescent DCF. In this experiment, the change in fluorescence intensity was quantified on an open scale (perceivable signal) comparing neuronal vs*.* non-neuronal areas. While CA1 pyramidal neurons exposed to 100 nmol/L Rot exhibited slight increases in baseline ROS levels for up to 25 min after the start of infusion (nine cells from three rats; Fig. 5a), overall, the values were not significantly different from the baseline. Furthermore, to exclude the possibility that 2 mmol/L K_2_ATP inside the pipette solution might mask the effect of rotenone, we measured ROS levels in CA1 hippocampal slices after bath incubation of DHE. Consistently, DHE stained cells exposed to 100 nmol/L Rot did not significantly alter ROS level for 50 min (Fig. 5b). In contrast, cells exposed to 1 μmol/L Rot exhibited significantly higher fluorescence intensities compared to both control and 100 nmol/L Rot-exposed groups (*p*< 0.05, *n*= 9). And, as predicted, 1 mmol/L 3-NP resulted in a further increase in fluorescence (*n*= 10) compared to 1 μmol/L Rot. The addition of ketones fully prevented this change (*n*= 11) (Fig. 5b).

### Ketones restored ATP depletion caused by H_2_O_2_ and MRC inhibition

**Figure 6:**
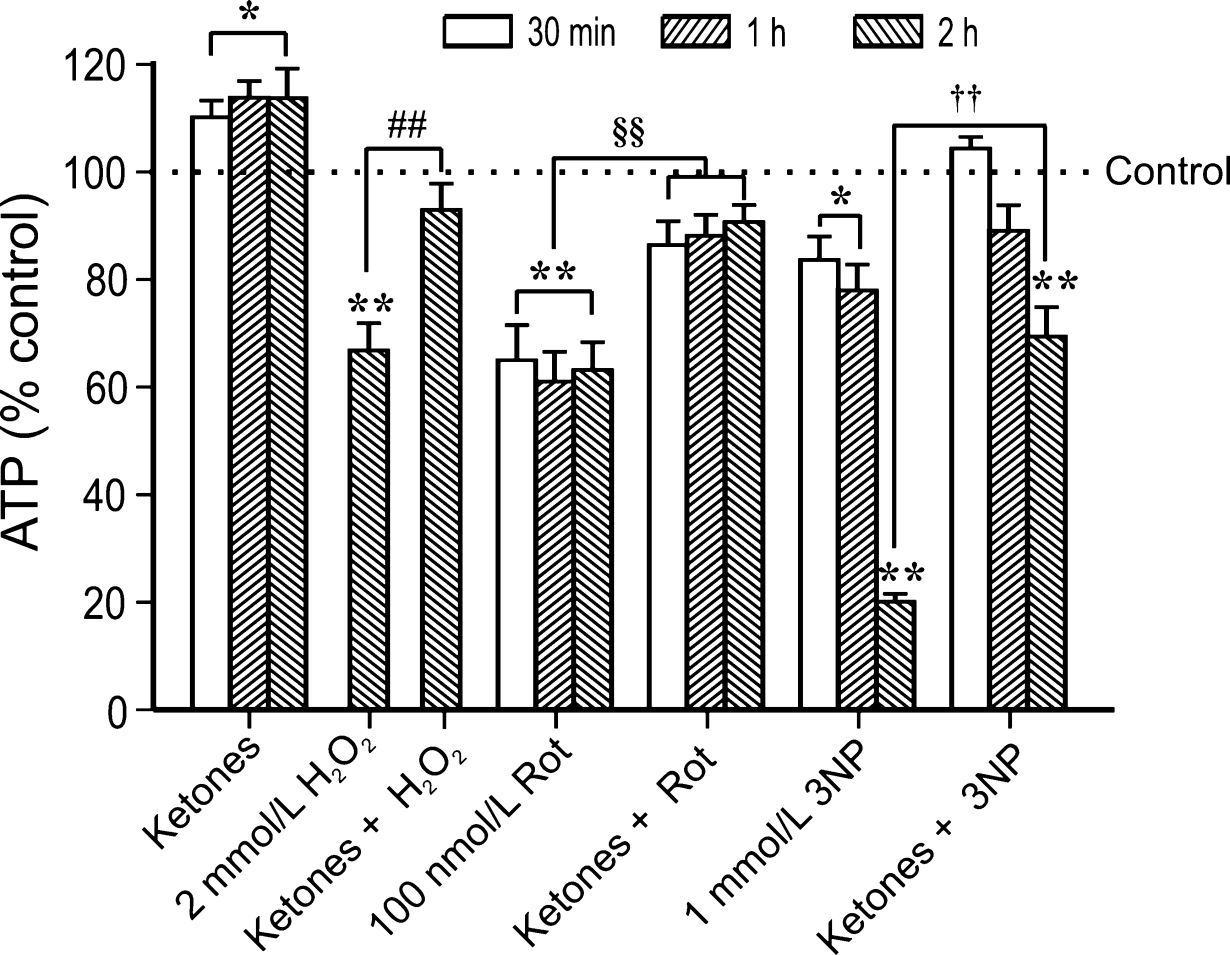
Ketones restore ATP levels depleted by oxidative stress and by inhibition of MRC function. ATP levels (represented as % of control) in micro-dissected CA1 tissue samples treated with H_2_O_2_, ketones plus H_2_O_2_, ketones, rotenone, ketones plus rotenone, 3-NP and ketones plus 3-NP are shown at various time-points. Control slices were perfused with physiological saline under similar experimental conditions. Values represent mean ± SEM. Paired *t*-tests were conducted in control *vs.* drug-treated groups; *, *p* < 0.05; **, *p* < 0.01: 2 mmol/L H_2_O_2_*vs.* ketones plus 2 mmol/L H_2_O_2_; ##, *p* < 0.05: 1 mmol/L 3-NP *vs.* ketones plus 1 mmol/L 3-NP; ††, *p* < 0.01. ANOVA followed by Tukey test; §§, *p* < 0.01.

While our data strongly indicated that ketone-induced synaptic protection against MRC dysfunction may partially involve an antioxidant mechanism, impairment of hippocampal synaptic transmission induced by Rot may not in fact be a consequence of ROS generation. In support of this, Tieu *et al.* (2003) reported that BHB exerted neuroprotective effects against Rot exposure in a model of Parkinson’s disease by increasing mitochondrial ATP production. As such, we then asked whether the protective effects of ketones against oxidative stress and MRC inhibition might correlate with ATP levels in the CA1 region of the hippocampus. Ketones produced a small but significant increase in ATP levels of micro-dissected CA1 tissue after exposure for 30 min, 1 h and 2 h (*p*< 0.05; Fig. 6). H_2_O_2_ (2 mmol/L) significantly reduced ATP levels to 67.0 ± 6.5% of baseline after 2-h exposure. Co-application of ketones with H_2_O_2_ restored ATP levels to 92.9 ± 4.8% of control. Values were significantly different from those obtained with H_2_O_2_ alone (*p*< 0.01), but not compared to control (Fig. 6). Rot (100 nmol/L) significantly decreased ATP levels to 64.9 ± 6.5%, 61.0 ± 5.5% and 63.1 ± 5% of baseline at 30 min, 1 h, and 2 h time-points, respectively (*p*< 0.01). Co-application of the ketones and Rot (100 nmol/L) resulted in a restoration of ATP levels, to 86.4 ± 4.4%, 88.1 ± 3.9% and 91.7 ± 3.2% of control levels at the three time-points, respectively (Fig. 6). Differences between control and Rot groups, and between Rot and ketones plus Rot groups were statistically significant (*p*< 0.01). However, there were no significant differences between the control and the ketones plus Rot groups (Fig. 6). 3-NP (1 mmol/L) also decreased ATP levels over similar incubation times; the ATP values were 83.6 ± 4.3%, 77.9 ± 4.8% and 30.0 ± 1.5% of control values, respectively. Notably, a 2-h application of 3-NP produced a dramatic decrease in ATP levels (*p*< 0.001). Conversely, application of ketones plus 3-NP restored ATP levels at two time-points (104 ± 2.1% and 89.1 ± 4.7% of controls at 30 min and after a 1-h exposure, respectively). Although a 2-h application of ketones plus 3-NP significantly increased ATP levels (to 69.3 ± 5.4% of control values; *p*< 0.001) in CA1 hippocampus compared to the 2-h 3-NP alone treatment group, ketone treatment alone also differed significantly from the controls (*p*< 0.01). Taken together, these data indicated that ketones may provide functional neuroprotection against MRC dysfunction via ATP restoration.

## Discussion

The principal findings of the current study are that: (i) MRC inhibitors impair hippocampal synaptic transmission; (ii) ketones protect against MRC impairment through restoration of catalase activity and ATP production; (iii) the dose-dependent protective action of ketones against complex I inhibition by Rot is ROS-independent; and (iv) the primary mechanism of acute Rot-induced synaptic suppression may involve ATP depletion. Together, our data suggest that ketones exert antioxidant activity and increase bioenergetic reserves, both of which serve to help maintain normal synaptic integrity in CA1 hippocampus in the face of MRC compromise.

### Inhibition of MRC I

It is well known that decreased activity of MRC I is linked to the pathogenesis of PD. MRC I inhibitors such as rotenone and MPP^+^ recapitulate the neuropathological features of PD both *in vivo* and *in vitro*. Such neuronal injury is thought to arise from suppression of electron transfer through MRC I, which subsequently triggers mitochondrial ROS production and ATP depletion (Sherer *et al.* 2003; Zhao *et al.* 2006). However, despite clear morphological data showing the effects of pathologically reduced MRC I activity, parallel evidence of cognitive dysfunction are notably lacking. In the present report, we provide strong evidence that impairment of MRC I results in attenuation of hippocampal synaptic transmission.

Our electrophysiological findings in hippocampus are consistent with a recent report demonstrating that rotenone causes a dose-dependent and irreversible loss of the field potential amplitude in striatal spiny neurons (Costa *et al.* 2008). With respect to the effects of Rot (100 nmol/L) on bioenergetic substrates, our data are also in line with a previous report demonstrating decreased ATP levels in spiny neurons induced by Rot (Bao *et al.* 2005). Moreover, when considered together with our ketone experiments, our data further support an earlier hypothesis that ketone-mediated protection against Rot-induced injury is associated with, and perhaps explained by, the presence of higher levels of bioenergetic substrates such as ATP (Tieu *et al.* 2003; Masuda *et al.* 2005; Zhao *et al.* 2006).

In contrast to earlier studies, however, we did not observe significant changes in ROS levels following Rot (100 nmol/L) application as determined by measurement of ROS (DCF)/superoxide (DHE) fluorescence. Specifically, cell-permeable antioxidants had no effect on hippocampal synaptic suppression induced by Rot. Although our results support the view that Rot-induced synaptic suppression is ROS-independent (Frantseva *et al.* 2001; Sherer *et al.* 2003), we cannot exclude the possibility that higher concentrations of Rot (1 μmol/L) might be necessary to raise ROS levels sufficiently to impair hippocampal synaptic transmission. Collectively, our data suggest that MRC I dysfunction by Rot can lead to dose-dependent synaptic suppression via both ATP-dependent and ROS-independent or dependent mechanisms.

### Inhibition of MRC II

Succinate dehydrogenase regulates the oxidation of succinate to fumarate, and as such, is a critical enzyme in the tricarboxylic acid cycle, in MRC function, and in maintaining cellular viability. In support of this, inhibition of succinate dehydrogenase activity by 3-NP induced neuronal death, likely caused by depletion of ATP (as a consequence of MRC II inhibition), oxidative stress, and excitotoxicity via calcium influx through NMDA receptors (Ayala *et al.* 2007). Furthermore, it has been shown that 3-NP decreases MRC II activity with regional and cell-type specificity within the brain (Calabresi *et al.* 2001). Intriguingly, however, systemic application of 3-NP preferentially induces CA1 hippocampal injury (Pang and Geddes 1997; Rodriguez-Martinez *et al.* 2004; Karanian *et al.* 2006).

In the present study, acute 3-NP application gradually decreased ATP levels within 1 h, and resulted in a more dramatic drop after 2 h. However, in contrast to our experiments with Rot, antioxidants reversed the suppression of the PS amplitude induced by 3-NP, consistent with increased ROS production. These results support previous studies demonstrating that antioxidants such as vitamin C, vitamin E, and taurine can mitigate 3-NP-induced neuronal injury in rat hippocampus (Rodriguez-Martinez *et al.* 2004; Montiel *et al.* 2006). Collectively, published data and the results of the present study strongly suggest that 3-NP-induced hippocampal synaptic suppression may occur in part through oxidative as well as metabolic stress.

### Mechanism of ketone-induced synaptic protection

Aberrant mitochondrial ROS production has been strongly implicated as a major trigger for pathogenesis of ND (Lin and Beal 2006; Mancuso *et al.* 2006). At low concentrations, ROS may play a critical role in normal synaptic function (Klann and Thiels 1999; Serrano and Klann 2004). However, when ROS generation overwhelms cellular antioxidant capacity, synaptic impairment and neuronal injury may ensue (Pellmar 1987; Avshalumov *et al.* 2000; Kamsler and Segal 2003). In support of this concept, extracellular H_2_O_2_ levels were highly elevated (to approximately 200 μM) after forebrain ischemic injury (Hyslop *et al.* 1995; Lei *et al.* 1997) and exogenous application of H_2_O_2_ provoked dose-dependent synaptic impairment (Pellmar 1987; Avshalumov *et al.* 2000).

In this context, our prior studies have shown that ketones exert neuronal/synaptic protection by diminishing oxidative stress, perhaps through an antioxidant action (Kim *et al.* 2007; Maalouf and Rho 2008). However, ketones *per se* may not exert direct antioxidant scavenging effects against H_2_O_2_, despite effective scavenging of hydroxyl radicals (with IC_50_ values for BHB and ACA of 3.2 and 31 mM, respectively) as reported by Haces *et al.* (2008). Indirect evidence for ketone-mediated antioxidant activity is evidenced by the fact that the high-fat anticonvulsant KD (which induces significant ketonemia) increases glutathione peroxidase activity and enhances mitochondrial redox status via increased glutathione in rat hippocampus (Ziegler *et al.* 2003; Jarrett *et al.* 2008). While it is unclear whether these findings are a direct result of ketone action, our observation that the suppression of catalase activity following oxidative stress can be reversed by ketones lends credence to this notion.

One outstanding issue is whether ketone-induced protective effects can be blocked by MRC II inhibition. In previous studies involving dopaminergic or spinal cord neurons, MRC II inhibitors prevented the beneficial effects of BHB – which has shown to improve mitochondrial respiration and ATP generation (Tieu *et al.* 2003; Zhao *et al.* 2006). However, in dopaminergic SH-SY5Y cells, a MRC II inhibitor failed to completely abolish the neuroprotective effect of BHB (Imamura *et al.* 2006). Not surprisingly, in the present study, ketones enhanced synaptic protection by preserving both the PS and field potential in the face of Rot exposure through enhanced ATP generation. Interestingly, the synaptic protective effects of ketones against 3-NP exposure exhibited regional specificity (even within the same hippocampus); ketones exerted differential effects on the evoked potential (i.e., population spike vs*.* field potential) and on brain region (stratum pyramidale vs*.* stratum radiatum). Although it is known that impairment of both PS and field potential amplitude *in vitro* by H_2_O_2_ in the hippocampus is dose- and site-specific (Pellmar 1987; Avshalumov *et al.* 2000), the underlying mechanisms for the variability seen in hippocampus remains unclear. Nevertheless, recovery of the PS amplitude after ketone application is more robust than that of the field potential when exposed to Rot or 3-NP. This discrepancy can be explained in part by ketone-induced restoration of ATP levels in CA1 hippocampus, which would contribute to enhanced PS amplitude against MRC dysfunction. Further, these differences may be related to region-specific differences in mitochondrial numbers (Palay 1956; Keating 2008).

Along similar lines, it has recently been shown that the KD (which induces ketonemia) exerts opposing region- (and possibly age-) specific effects in the aging hippocampus (i.e., CA1 vs. dentate gyrus) (Balietti *et al.* 2008). However, age may be an important determinant of the neuroprotection afforded by the KD (and possibly ketones), as the KD rendered protective effects against traumatic brain injury in immature rodents but not in aged animals (Appelberg *et al.* 2009). Although the differential functional effects of the KD in the aged brain have yet to be elucidated, our present findings strongly support the view that ketones consistently provide synaptic protective effects in the younger brain when challenged by MRC dysfunction. In the end, the synaptic protective mechanisms of ketones against 3-NP neurotoxicity are likely complex, but appear to be related to the enhancement of antioxidant capacity and increases in ATP levels.

### Other potential mechanisms

There are several lines of experimental evidence suggesting that either up-regulation or restoration of bioenergetic capacity in mitochondria may underlie the neuroprotective and anticonvulsant effects of adenosine, which has previously been shown to maintain normal synaptic functioning (Masino and Geiger 2008). Notwithstanding the lack of direct evidence of purinergic modulation by ketones, it is conceivable that higher ATP levels induced by ketones may result in enhanced modulation of adenosine A_1_ or A_2A_ receptors. While activation of these receptor subtypes may lead to opposing actions at the level of the hippocampal synapse (Rebola *et al.* 2008; Liang *et al.* 2009; Serpa *et al.* 2009), further study involving the functional inter-relationships between adenosine receptor activation and ketones will undoubtedly advance our mechanistic understanding of ketone-induced synaptic protection.

Another potential mechanism of ketone-induced neuroprotection is through restoration of Na^+^-K^+^-ATPase function. When the MRC is disrupted, decreased energy supply diminishes Na^+^-K^+^-ATPase activity, thereby destabilizing the resting membrane potential. In support of this, exogenous application of ATP suppressed Rot-induced increases in NMDA currents, whereas this effect was reversed by strophanthidin, ATP-independent sodium channel blocker, suggesting that ATP depletion results in the disrupted membrane potential (Wu and Johnson 2007).

One of the intriguing findings in the present study is that MRC dysfunction strongly reduced ATP levels even in the presence of 10 mmol/L glucose, indicating that ATP derived solely from glycolysis cannot fully compensate for overall cellular ATP demands. Consistent with the protective effect of exogenous ATP application against MRC inhibition (Wu and Johnson 2007), both the present study and previous reports have shown that ketones and the full KD enable conditions of up-regulated ATP synthesis through multiple mechanisms that normalize MRC activity and hence stabilize the mitochondrial inner membrane potential (Maalouf *et al.* 2009). For example, we have previously shown that ketones enhance NADH oxidation (Maalouf *et al.* 2007). Further, Zhao *et al.* (2006) demonstrated that BHB was able to prevent rotenone-induced inhibition of MRC I (but not malonate inhibition of MRC II), and promoted mitochondrial ATP synthesis. On another level, the KD further enhances mitochondrial biogenesis which would be consistent with overall increases in ATP production (Bough *et al.* 2006; Nylen *et al.* 2009). Thus, separate from ongoing glycolytic metabolism – as would be expected under the hippocampal slice recording conditions used in the present study (with 10 mmol/L glucose) – it is clear that functional synaptic integrity depends upon normal ATP synthesis in mitochondria, and that ketones exert protective effects against MRC impairment, likely through enhanced mitochondrial ATP production and diminution of ROS.

### Clinical relevance

It is well known that MRC dysfunction is a critical hallmark of certain ND (Petrozzi *et al.* 2007). Huntington’s disease, characterized clinically by bradykinesia, cognitive impairment, and chorea, has been linked to impaired MRC II function (Alexi *et al.* 1998). In AD transgenic mice, mitochondrial dysfunction, along with amyloid precursor protein-induced ROS generation, results in increased energy demands (Takuma *et al.* 2005). Similarly, secondary injury following traumatic brain injury may arise from mitochondrial dysfunction, which increases oxidative stress, specifically through facilitation of ROS generation and ATP depletion (Sullivan *et al.* 2005; Opii *et al.* 2007).

Thus, several attempts to elucidate underlying mechanisms of ND have addressed disrupted mitochondrial ROS/ATP generation resulting from MRC dysfunction (Alexi *et al.* 1998; Lin and Beal 2006; Mancuso *et al.* 2006), and there is increasing speculation that mitochondrial ROS and ATP might play a role in modulating synaptic transmission (Keating 2008). Importantly, the results of the present study link functional synaptic integrity with perturbations in MRC function, and highlight the protective role that ketones play in this regard.

## Summary

In the present study, we have shown that ketones help maintain synaptic transmission against oxidative stress, believed to be a common pathogenic mechanism involved in neurodegenerative disorders. Our data suggest that the antioxidant activity of ketones may play an important role in the restoration of synaptic transmission in the face of oxidative injury, and that this protective effect may involve enhanced antioxidant activity and ATP synthesis. With the expanding evidence that ketones exert synaptic protective effects through a number of mechanisms operant at the level of the mitochondrion, there is now a timely opportunity to assess the validity of this novel therapeutic intervention for a variety of neurodegenerative disorders (Smith *et al.* 2005; Henderson *et al.* 2009).

## Abbreviations

3-NP: 3-nitropropionic acid
ACA: acetoacetate
AD: Alzheimer’s disease
BHB: (R)-(−)-3-hydroxybutyric acid
CA: cornu Ammonis
DHE: dihydroethidium
DMSO: dimethylsulfoxide
GSHMEE: glutathione monoester
H_2_-DCFDA: 2′,7′-dichlorofluorescein diacetate
KD: ketogenic diet
MnTBAP: Mn(III) tetrakis (4-benzoic acid) porphyrin
MRC: mitochondrial respiratory complex
ND: neurodegenerative diseases
PD: Parkinson’s disease
PS: population spike
ROS: reactive oxygen species
Rot: rotenone

## References

[b1] Alexi T, Hughes PE, Faull RL, Williams CE (1998). 3-Nitropropionic acid’s lethal triplet: cooperative pathways of neurodegeneration. Neuroreport.

[b2] Appelberg KS, Hovda DA, Prins ML (2009). The effects of a ketogenic diet on behavioral outcome after controlled cortical impact injury in the juvenile and adult rat. J. Neurotrauma.

[b3] Avshalumov MV, Chen BT, Rice ME (2000). Mechanisms underlying H(2)O(2)-mediated inhibition of synaptic transmission in rat hippocampal slices. Brain Res.

[b4] Ayala A, Venero JL, Cano J, Machado A (2007). Mitochondrial toxins and neurodegenerative diseases. Front. Biosci.

[b5] Balietti M, Giorgetti B, Fattoretti P (2008). Ketogenic diets cause opposing changes in synaptic morphology in CA1 hippocampus and dentate gyrus of late-adult rats. Rejuvenation Res.

[b6] Bao L, Avshalumov MV, Rice ME (2005). Partial mitochondrial inhibition causes striatal dopamine release suppression and medium spiny neuron depolarization via H2O2 elevation, not ATP depletion. J. Neurosci.

[b7] Baud O, Greene AE, Li J, Wang H, Volpe JJ, Rosenberg PA (2004). Glutathione peroxidase-catalase cooperativity is required for resistance to hydrogen peroxide by mature rat oligodendrocytes. J. Neurosci.

[b8] Bough KJ, Wetherington J, Hassel B, Pare JF, Gawryluk JW, Greene JG, Shaw R, Smith Y, Geiger JD, Dingledine RJ (2006). Mitochondrial biogenesis in the anticonvulsant mechanisms of the ketogenic diet. Ann. Neurol.

[b9] Calabresi P, Gubellini P, Picconi B, Centonze D, Pisani A, Bonsi P, Greengard P, Hipskind RA, Borrelli E, Bernardi G (2001). Inhibition of mitochondrial complex II induces a long-term potentiation of NMDA-mediated synaptic excitation in the striatum requiring endogenous dopamine. J. Neurosci.

[b10] Cassarino DS, Bennett JP (1999). An evaluation of the role of mitochondria in neurodegenerative diseases: mitochondrial mutations and oxidative pathology, protective nuclear responses, and cell death in neurodegeneration. Brain Res. Brain Res. Rev.

[b11] Costa C, Belcastro V, Tozzi A (2008). Electrophysiology and pharmacology of striatal neuronal dysfunction induced by mitochondrial complex I inhibition. J. Neurosci.

[b12] Dardzinski BJ, Smith SL, Towfighi J, Williams GD, Vannucci RC, Smith MB (2000). Increased plasma beta-hydroxybutyrate, preserved cerebral energy metabolism, and amelioration of brain damage during neonatal hypoxia ischemia with dexamethasone pretreatment. Pediatr. Res.

[b13] Davies S, Ramsden DB (2001). Huntington’s disease. Mol. Pathol.

[b14] Foster KA, Galeffi F, Gerich FJ, Turner DA, Muller M (2006). Optical and pharmacological tools to investigate the role of mitochondria during oxidative stress and neurodegeneration. Prog. Neurobiol.

[b15] Frantseva MV, Carlen PL, Perez Velazquez JL (2001). Dynamics of intracellular calcium and free radical production during ischemia in pyramidal neurons. Free Radic. Biol. Med.

[b16] Haces ML, Hernandez-Fonseca K, Medina-Campos ON, Montiel T, Pedraza-Chaverri J, Massieu L (2008). Antioxidant capacity contributes to protection of ketone bodies against oxidative damage induced during hypoglycemic conditions. Exp. Neurol.

[b17] Henderson ST, Vogel JL, Barr LJ, Garvin F, Jones JJ, Costantini LC (2009). Study of the ketogenic agent AC-1202 in mild to moderate Alzheimer’s disease: a randomized, double-blind, placebo-controlled, multicenter trial. Nutr. Metab. (Lond).

[b18] Huber SJ, Shuttleworth EC, Paulson GW (1986). Dementia in Parkinson’s disease. Arch. Neurol.

[b19] Hyslop PA, Zhang Z, Pearson DV, Phebus LA (1995). Measurement of striatal H2O2 by microdialysis following global forebrain ischemia and reperfusion in the rat: correlation with the cytotoxic potential of H2O2 in vitro. Brain Res.

[b20] Imamura K, Takeshima T, Kashiwaya Y, Nakaso K, Nakashima K (2006). D-beta-hydroxybutyrate protects dopaminergic SH-SY5Y cells in a rotenone model of Parkinson’s disease. J. Neurosci. Res.

[b21] Izumi Y, Ishii K, Katsuki H, Benz AM, Zorumski CF (1998). beta-Hydroxybutyrate fuels synaptic function during development. Histological and physiological evidence in rat hippocampal slices. J. Clin. Invest.

[b22] Jarrett SG, Milder JB, Liang LP, Patel M (2008). The ketogenic diet increases mitochondrial glutathione levels. J. Neurochem.

[b23] Kamsler A, Segal M (2003). Hydrogen peroxide modulation of synaptic plasticity. J. Neurosci.

[b24] Kang HC, Lee YM, Kim HD, Lee JS, Slama A (2007). Safe and effective use of the ketogenic diet in children with epilepsy and mitochondrial respiratory chain complex defects. Epilepsia.

[b25] Karanian DA, Baude AS, Brown QB, Parsons CG, Bahr BA (2006). 3-Nitropropionic acid toxicity in hippocampus: protection through N-methyl-D-aspartate receptor antagonism. Hippocampus.

[b26] Kashiwaya Y, Takeshima T, Mori N, Nakashima K, Clarke K, Veech RL (2000). D-beta-hydroxybutyrate protects neurons in models of Alzheimer’s and Parkinson’s disease. Proc. Natl Acad. Sci. USA.

[b27] Keating DJ (2008). Mitochondrial dysfunction, oxidative stress, regulation of exocytosis and their relevance to neurodegenerative diseases. J. Neurochem.

[b28] Kim do Y, Rho JM (2008). The ketogenic diet and epilepsy. Curr. Opin. Clin. Nutr. Metab. Care.

[b29] Kim DY, Davis LM, Sullivan PG, Maalouf M, Simeone TA, Brederode JV, Rho JM (2007). Ketone bodies are protective against oxidative stress in neocortical neurons. J. Neurochem.

[b30] Klann E, Thiels E (1999). Modulation of protein kinases and protein phosphatases by reactive oxygen species: implications for hippocampal synaptic plasticity. Prog. Neuropsychopharmacol. Biol. Psychiatry.

[b31] Kudin AP, Bimpong-Buta NY, Vielhaber S, Elger CE, Kunz WS (2004). Characterization of superoxide-producing sites in isolated brain mitochondria. J. Biol. Chem.

[b32] Kweon GR, Marks JD, Krencik R, Leung EH, Schumacker PT, Hyland K, Kang UJ (2004). Distinct mechanisms of neurodegeneration induced by chronic complex I inhibition in dopaminergic and non-dopaminergic cells. J. Biol. Chem.

[b33] Lei B, Adachi N, Arai T (1997). The effect of hypothermia on H2O2 production during ischemia and reperfusion: a microdialysis study in the gerbil hippocampus. Neurosci. Lett.

[b34] Liang R, Pang ZP, Deng P, Xu ZC (2009). Transient enhancement of inhibitory synaptic transmission in hippocampal CA1 pyramidal neurons after cerebral ischemia. Neuroscience.

[b35] Lin MT, Beal MF (2006). Mitochondrial dysfunction and oxidative stress in neurodegenerative diseases. Nature.

[b36] Maalouf M, Rho JM (2008). Oxidative impairment of hippocampal long-term potentiation involves activation of protein phosphatase 2A and is prevented by ketone bodies. J. Neurosci. Res.

[b37] Maalouf M, Sullivan PG, Davis L, Kim DY, Rho JM (2007). Ketones inhibit mitochondrial production of reactive oxygen species production following glutamate excitotoxicity by increasing NADH oxidation. Neuroscience.

[b38] Maalouf M, Rho JM, Mattson MP (2009). The neuroprotective properties of calorie restriction, the ketogenic diet, and ketone bodies. Brain Res. Rev.

[b39] Mancuso M, Coppede F, Migliore L, Siciliano G, Murri L (2006). Mitochondrial dysfunction, oxidative stress and neurodegeneration. J. Alzheimers Dis.

[b40] Masino SA, Geiger JD (2008). Are purines mediators of the anticonvulsant/neuroprotective effects of ketogenic diets?. Trends Neurosci.

[b41] Masuda R, Monahan JW, Kashiwaya Y (2005). D-beta-hydroxybutyrate is neuroprotective against hypoxia in serum-free hippocampal primary cultures. J. Neurosci. Res.

[b42] Montiel T, Quiroz-Baez R, Massieu L, Arias C (2006). Role of oxidative stress on beta-amyloid neurotoxicity elicited during impairment of energy metabolism in the hippocampus: protection by antioxidants. Exp. Neurol.

[b43] Nordli DR, De Vivo DC (1997). The ketogenic diet revisited: back to the future. Epilepsia.

[b44] Nylen K, Velazquez JL, Sayed V, Gibson KM, Burnham WM, Snead OC (2009). The effects of a ketogenic diet on ATP concentrations and the number of hippocampal mitochondria in Aldh5a1(-/-) mice. Biochim. Biophys. Acta.

[b45] Opii WO, Nukala VN, Sultana R, Pandya JD, Day KM, Merchant ML, Klein JB, Sullivan PG, Butterfield DA (2007). Proteomic identification of oxidized mitochondrial proteins following experimental traumatic brain injury. J. Neurotrauma.

[b46] Palay SL (1956). Synapses in the central nervous system. J. Biophys. Biochem. Cytol.

[b47] Pang Z, Geddes JW (1997). Mechanisms of cell death induced by the mitochondrial toxin 3-nitropropionic acid: acute excitotoxic necrosis and delayed apoptosis. J. Neurosci.

[b48] Panov A, Dikalov S, Shalbuyeva N, Taylor G, Sherer T, Greenamyre JT (2005). Rotenone model of Parkinson disease: multiple brain mitochondria dysfunctions after short term systemic rotenone intoxication. J. Biol. Chem.

[b49] Pellmar TC (1987). Peroxide alters neuronal excitability in the CA1 region of guinea-pig hippocampus in vitro. Neuroscience.

[b50] Petrozzi L, Ricci G, Giglioli NJ, Siciliano G, Mancuso M (2007). Mitochondria and neurodegeneration. Biosci. Rep.

[b51] Prins ML (2008). Cerebral metabolic adaptation and ketone metabolism after brain injury. J. Cereb. Blood Flow Metab.

[b52] Rebola N, Lujan R, Cunha RA, Mulle C (2008). Adenosine A2A receptors are essential for long-term potentiation of NMDA-EPSCs at hippocampal mossy fiber synapses. Neuron.

[b53] Rodriguez-Martinez E, Rugerio-Vargas C, Rodriguez AI, Borgonio-Perez G, Rivas-Arancibia S (2004). Antioxidant effects of taurine, vitamin C, and vitamin E on oxidative damage in hippocampus caused by the administration of 3-nitropropionic acid in rats. Int. J. Neurosci.

[b54] Saybasili H, Yuksel M, Haklar G, Yalcin AS (2001). Effect of mitochondrial electron transport chain inhibitors on superoxide radical generation in rat hippocampal and striatal slices. Antioxid. Redox Signal.

[b55] Serpa A, Ribeiro JA, Sebastiao AM (2009). Cannabinoid CB(1) and adenosine A(1) receptors independently inhibit hippocampal synaptic transmission. Eur. J. Pharmacol.

[b56] Serrano F, Klann E (2004). Reactive oxygen species and synaptic plasticity in the aging hippocampus. Ageing Res. Rev.

[b57] Sherer TB, Betarbet R, Testa CM, Seo BB, Richardson JR, Kim JH, Miller GW, Yagi T, Matsuno-Yagi A, Greenamyre JT (2003). Mechanism of toxicity in rotenone models of Parkinson’s disease. J. Neurosci.

[b58] Smith SL, Heal DJ, Martin KF (2005). KTX 0101: a potential metabolic approach to cytoprotection in major surgery and neurological disorders. CNS Drug Rev.

[b59] Sullivan PG, Rabchevsky AG, Waldmeier PC, Springer JE (2005). Mitochondrial permeability transition in CNS trauma: cause or effect of neuronal cell death?. J. Neurosci. Res.

[b60] Suzuki M, Sato K, Dohi S, Sato T, Matsuura A, Hiraide A (2001). Effect of beta-hydroxybutyrate, a cerebral function improving agent, on cerebral hypoxia, anoxia and ischemia in mice and rats. Jpn. J. Pharmacol.

[b61] Takuma K, Yao J, Huang J, Xu H, Chen X, Luddy J, Trillat AC, Stern DM, Arancio O, Yan SS (2005). ABAD enhances Abeta-induced cell stress via mitochondrial dysfunction. FASEB J.

[b62] Thio LL, Wong M, Yamada KA (2000). Ketone bodies do not directly alter excitatory or inhibitory hippocampal synaptic transmission. Neurology.

[b63] Tieu K, Perier C, Caspersen C (2003). D-beta-hydroxybutyrate rescues mitochondrial respiration and mitigates features of Parkinson disease. J. Clin. Invest.

[b64] Veech RL (2004). The therapeutic implications of ketone bodies: the effects of ketone bodies in pathological conditions: ketosis, ketogenic diet, redox states, insulin resistance, and mitochondrial metabolism. Prostaglandins Leukot. Essent. Fatty Acids.

[b65] Veech RL, Chance B, Kashiwaya Y, Lardy HA, Cahill GF (2001). Ketone bodies, potential therapeutic uses. IUBMB Life.

[b66] Wu YN, Johnson SW (2007). Rotenone potentiates NMDA currents in substantia nigra dopamine neurons. Neurosci. Lett.

[b67] Zhao Z, Lange DJ, Voustianiouk A, MacGrogan D, Ho L, Suh J, Humala N, Thiyagarajan M, Wang J, Pasinetti GM (2006). A ketogenic diet as a potential novel therapeutic intervention in amyotrophic lateral sclerosis. BMC Neurosci.

[b68] Ziegler DR, Ribeiro LC, Hagenn M, Siqueira IR, Araujo E, Torres IL, Gottfried C, Netto CA, Goncalves CA (2003). Ketogenic diet increases glutathione peroxidase activity in rat hippocampus. Neurochem. Res.

